# 6-(3-Pyrid­yl)-3-(3,4,5-trimethoxy­phen­yl)-1,2,4-triazolo[3,4-*b*][1,3,4]thia­diazole

**DOI:** 10.1107/S1600536808019855

**Published:** 2008-07-05

**Authors:** Haitang Du, Haijun Du, Ying An, Shengnan Li

**Affiliations:** a Department of Biology and Environment Technology, Guiyang College, Guiyang 550005, People’s Republic of China; b School of Chemistry and Environment Science, Guizhou University for Nationalities, Guiyang 550025, People’s Republic of China; c Department of Chemistry, College of Science, Tianjin University, Tianjin 300072, People’s Republic of China

## Abstract

In the mol­ecule of the title compound, C_17_H_15_N_5_O_3_S, the planar central heterocylic ring system is oriented with respect to the benzene and pyridine rings at dihedral angles of 6.61 (3) and 19.22 (3)°, respectively. An intra­molecular C—H⋯N hydrogen bond results in the formation of a six-membered ring, adopting a flattened boat conformation. In the crystal structure, inter­molecular C—H⋯N hydrogen bonds link the mol­ecules.

## Related literature

For general background, see: Karabasanagouda *et al.* (2007 ▶); Mathew *et al.* (2007 ▶). For ring conformation puckering parameters, see: Cremer & Pople (1975 ▶).
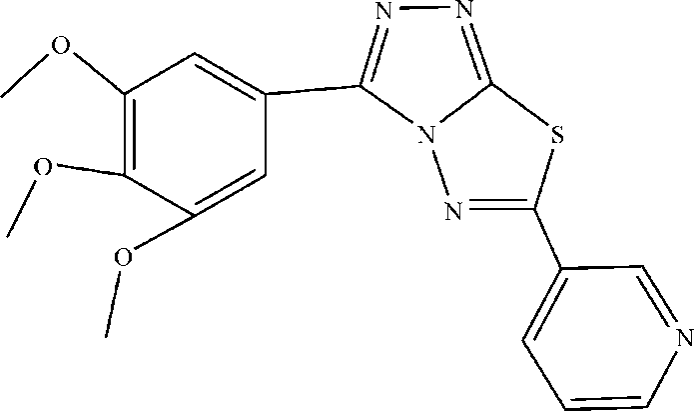


## Experimental

### 

#### Crystal data

C_17_H_15_N_5_O_3_S
*M* *_r_* = 369.40
Monoclinic, 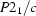

*a* = 7.4682 (15) Å
*b* = 14.128 (3) Å
*c* = 15.550 (3) Å
β = 90.46 (3)°
*V* = 1640.6 (6) Å^3^
*Z* = 4
Mo *K*α radiation
μ = 0.23 mm^−1^
*T* = 113 (2) K
0.20 × 0.06 × 0.04 mm

#### Data collection

Rigaku Saturn CCD area-detector diffractometer
Absorption correction: multi-scan (*CrystalClear*; Rigaku/MSC, 2005 ▶) *T*_min_ = 0.956, *T*_max_ = 0.991
18716 measured reflections
3620 independent reflections
3121 reflections with *I* > 2σ(*I*)
*R*_int_ = 0.034

#### Refinement

*R*[*F* ^2^ > 2σ(*F* ^2^)] = 0.032
*wR*(*F* ^2^) = 0.108
*S* = 1.17
3620 reflections
238 parameters
H-atom parameters constrained
Δρ_max_ = 0.48 e Å^−3^
Δρ_min_ = −0.41 e Å^−3^

### 

Data collection: *CrystalClear* (Rigaku/MSC, 2005 ▶); cell refinement: *CrystalClear*; data reduction: *CrystalStructure* (Rigaku/MSC, 2005 ▶); program(s) used to solve structure: *SHELXS97* (Sheldrick, 2008 ▶); program(s) used to refine structure: *SHELXL97* (Sheldrick, 2008 ▶); molecular graphics: *SHELXTL* (Sheldrick, 2008 ▶); software used to prepare material for publication: *SHELXTL*.

## Supplementary Material

Crystal structure: contains datablocks I, global. DOI: 10.1107/S1600536808019855/hk2481sup1.cif

Structure factors: contains datablocks I. DOI: 10.1107/S1600536808019855/hk2481Isup2.hkl

Additional supplementary materials:  crystallographic information; 3D view; checkCIF report

## Figures and Tables

**Table 1:** Hydrogen-bond geometry (Å, °)

*D*—H⋯*A*	*D*—H	H⋯*A*	*D*⋯*A*	*D*—H⋯*A*
C2—H2⋯N4	0.95	2.40	3.0869 (19)	129
C9—H9*A*⋯N1^i^	0.98	2.60	3.576 (2)	171
C8—H8*C*⋯N5^ii^	0.98	2.63	3.573 (2)	161
C14—H14⋯N2^iii^	0.95	2.57	3.410 (2)	148

Symmetry codes: (i) 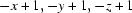; (ii) 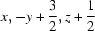; (iii) 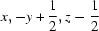.

**Fig. 1.**
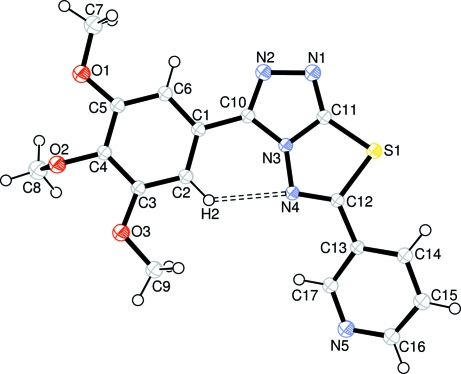
The molecular structure of the title molecule, with the atom-numbering scheme. Hydrogen bond is shown as dashed line.

## supplementary crystallographic information

### Comment

1,2,4-Triazole and 1,3,4-thiadiazole represent one of the most biologically active classes of compounds, possessing a wide spectrum of activities. Various substituted 1,2,4-triazolo[3,4-b]-1,3,4-thiadiazoles are associated with diverse pharmacological activities such as antimicrobial (Karabasanagouda *et al.*,  2007) and anti-inflammatory activity (Mathew *et al.*,  2007). We report herein the crystal structure of the title compound.

In the molecule of the title compound (Fig. 1) the bond lengths and angles are within normal ranges. Rings A (C1–C6), B (N1–N3/C10/C11), C (S1/N3/N4/C11/C12) and D (N5/C13–C17) are, of course, planar, and the dihedral angles between them are A/B = 6.28 (3)°, A/C = 6.97 (3)°, A/D = 25.30 (3)°, B/C = 0.95 (2)°, B/D = 19.38 (3)° and C/D = 19.06 (3)°. So, rings B and C are nearly coplanar. The coplanar ring system is oriented with respect to rings A and D at dihedral angles of 6.61 (3)° and 19.22 (3)°. The intramolecular C—H···N hydrogen bond (Table 1) results in the formation of a six-membered ring E (N3/N4/C1/C2/C10/H2), in which it adopts flattened-boat [φ = -95.41 (2)° and θ = 21.96 (3)°] conformation, having total puckering amplitude, Q_T_, of 1.463 (3) Å (Cremer & Pople, 1975).

In the crystal structure, intermolecular C—H···N hydrogen bonds (Table 1) link the molecules, in which they may be effective in the stabilization of the structure.

### Experimental

For the preparation of the title compound, 4-amino-5-(3,4,5-trimethoxyphenyl)-4H-1,2,4-triazole-3-thiol (0.01 M) and nicotinic acid (0.01 M) were dissolved in dry phosphorous oxychloride (10 ml). The resulted solution was further heated under reflux for 7 h. The reaction mixture was cooled to room temperature and the mixture was gradually poured onto crushed ice with stirring. Finally, powdered potassium carbonate and the required amount of solid potassium hydroxide were added until the pH of the mixture was raised to 8, to remove the excess of phosphorous oxychloride. The mixture was allowed to stand overnight and the solid was separated. It was filtered, washed with cold water, and then dried. Crystals suitable for X-ray analysis were obtained by the recrystallization of the solid residue from a mixture of N,N-dimethylformamide/ethanol (1:1) by slow evaporation at room temperature.

### Refinement

H atoms were positioned geometrically, with C—H = 0.95 and 0.98 Å for aromatic and methyl H, respectively, and constrained to ride on their parent atoms with U_iso_(H) = xU_eq_(C), where x = 1.5 for methyl H and x = 1.2 for aromatic H atoms.

### Crystal data

**Table:**

C_17_H_15_N_5_O_3_S	*F*_000_ = 768
*M**_r_* = 369.40	*D*_x_ = 1.496 Mg m^−^^3^
Monoclinic, *P*2_1_/*c*	Melting point: 448K K
Hall symbol: -P 2ybc	Mo *K*α radiation λ = 0.71073 Å
*a* = 7.4682 (15) Å	Cell parameters from 4807 reflections
*b* = 14.128 (3) Å	θ = 1.9–27.1º
*c* = 15.550 (3) Å	µ = 0.23 mm^−^^1^
β = 90.46 (3)º	*T* = 113 (2) K
*V* = 1640.6 (6) Å^3^	Prism, colourless
*Z* = 4	0.20 × 0.06 × 0.04 mm

### Data collection

**Table:**

Rigaku Saturn CCD area-detector diffractometer	3620 independent reflections
Radiation source: rotating anode	3121 reflections with *I* > 2σ(*I*)
Monochromator: confocal	*R*_int_ = 0.034
Detector resolution: 7.31 pixels mm^-1^	θ_max_ = 27.2º
*T* = 113(2) K	θ_min_ = 2.0º
φ and ω scans	*h* = −9→9
Absorption correction: multi-scan(CrystalClear; Rigaku/MSC, 2005)	*k* = −18→18
*T*_min_ = 0.956, *T*_max_ = 0.991	*l* = −19→19
18716 measured reflections	

### Refinement

**Table:**

Refinement on *F*^2^	Secondary atom site location: difference Fourier map
Least-squares matrix: full	Hydrogen site location: inferred from neighbouring sites
*R*[*F*^2^ > 2σ(*F*^2^)] = 0.032	H-atom parameters constrained
*wR*(*F*^2^) = 0.108	*w* = 1/[σ^2^(*F*_o_^2^) + (0.0643*P*)^2^ + 0.1727*P*] where *P* = (*F*_o_^2^ + 2*F*_c_^2^)/3
*S* = 1.17	(Δ/σ)_max_ = 0.001
3620 reflections	Δρ_max_ = 0.48 e Å^−^^3^
238 parameters	Δρ_min_ = −0.41 e Å^−^^3^
Primary atom site location: structure-invariant direct methods	Extinction correction: none

### Special details

**Table:**

Geometry. All e.s.d.'s (except the e.s.d. in the dihedral angle between two l.s. planes) are estimated using the full covariance matrix. The cell e.s.d.'s are taken into account individually in the estimation of e.s.d.'s in distances, angles and torsion angles; correlations between e.s.d.'s in cell parameters are only used when they are defined by crystal symmetry. An approximate (isotropic) treatment of cell e.s.d.'s is used for estimating e.s.d.'s involving l.s. planes.
Refinement. Refinement of F^2^ against ALL reflections. The weighted R-factor wR and goodness of fit S are based on F^2^, conventional R-factors R are based on F, with F set to zero for negative F^2^. The threshold expression of F^2^ > 2sigma(F^2^) is used only for calculating R-factors(gt) etc. and is not relevant to the choice of reflections for refinement. R-factors based on F^2^ are statistically about twice as large as those based on F, and R- factors based on ALL data will be even larger.

### Fractional atomic coordinates and isotropic or equivalent isotropic displacement parameters (Å^2^)

**Table:**

	*x*	*y*	*z*	*U*_iso_*/*U*_eq_	
S1	0.47268 (5)	0.30203 (2)	0.32685 (2)	0.01906 (12)	
O1	0.09766 (15)	0.49470 (8)	0.82026 (7)	0.0250 (3)	
O2	−0.02336 (14)	0.65367 (8)	0.74798 (7)	0.0239 (3)	
O3	0.02728 (14)	0.69219 (7)	0.58349 (7)	0.0236 (3)	
N1	0.46168 (17)	0.25227 (9)	0.50417 (8)	0.0228 (3)	
N2	0.39750 (17)	0.30533 (9)	0.57349 (8)	0.0217 (3)	
N3	0.36415 (15)	0.39115 (8)	0.45790 (8)	0.0167 (3)	
N4	0.32966 (15)	0.45519 (8)	0.39312 (7)	0.0167 (3)	
N5	0.32739 (18)	0.61126 (9)	0.16139 (9)	0.0242 (3)	
C1	0.24914 (18)	0.45989 (10)	0.59768 (9)	0.0178 (3)	
C2	0.18824 (19)	0.54403 (10)	0.56074 (9)	0.0183 (3)	
H2	0.2085	0.5569	0.5017	0.022*	
C3	0.09733 (18)	0.60873 (10)	0.61183 (9)	0.0184 (3)	
C4	0.07026 (18)	0.59006 (10)	0.69890 (9)	0.0186 (3)	
C5	0.13206 (18)	0.50562 (11)	0.73504 (9)	0.0192 (3)	
C6	0.22174 (18)	0.43975 (10)	0.68450 (9)	0.0189 (3)	
H6	0.2636	0.3820	0.7087	0.023*	
C7	0.1537 (2)	0.40859 (12)	0.85975 (10)	0.0285 (4)	
H7A	0.0953	0.3551	0.8309	0.043*	
H7B	0.1206	0.4092	0.9206	0.043*	
H7C	0.2839	0.4023	0.8549	0.043*	
C8	0.0881 (2)	0.72143 (13)	0.78883 (11)	0.0321 (4)	
H8A	0.1680	0.6895	0.8298	0.048*	
H8B	0.0139	0.7674	0.8194	0.048*	
H8C	0.1596	0.7543	0.7455	0.048*	
C9	0.0629 (2)	0.71792 (11)	0.49656 (10)	0.0240 (3)	
H9A	0.1922	0.7258	0.4890	0.036*	
H9B	0.0019	0.7776	0.4830	0.036*	
H9C	0.0190	0.6681	0.4580	0.036*	
C10	0.33868 (18)	0.38808 (10)	0.54526 (9)	0.0176 (3)	
C11	0.43862 (19)	0.30623 (10)	0.43654 (10)	0.0183 (3)	
C12	0.38009 (18)	0.41649 (10)	0.32131 (9)	0.0166 (3)	
C13	0.36482 (18)	0.46502 (10)	0.23842 (9)	0.0171 (3)	
C14	0.37053 (19)	0.41517 (10)	0.16155 (9)	0.0207 (3)	
H14	0.3865	0.3485	0.1614	0.025*	
C15	0.3526 (2)	0.46451 (11)	0.08527 (10)	0.0237 (3)	
H15	0.3555	0.4322	0.0317	0.028*	
C16	0.3304 (2)	0.56120 (11)	0.08802 (10)	0.0234 (3)	
H16	0.3166	0.5943	0.0352	0.028*	
C17	0.34453 (19)	0.56304 (10)	0.23455 (10)	0.0207 (3)	
H17	0.3428	0.5973	0.2871	0.025*	

### Atomic displacement parameters (Å^2^)

**Table:**

	*U*^11^	*U*^22^	*U*^33^	*U*^12^	*U*^13^	*U*^23^
S1	0.0235 (2)	0.01467 (19)	0.0190 (2)	0.00266 (13)	0.00292 (15)	−0.00077 (12)
O1	0.0308 (6)	0.0273 (6)	0.0169 (5)	0.0015 (5)	0.0062 (4)	0.0028 (4)
O2	0.0222 (5)	0.0234 (6)	0.0262 (6)	0.0008 (4)	0.0076 (4)	−0.0053 (4)
O3	0.0308 (6)	0.0189 (5)	0.0211 (6)	0.0049 (4)	0.0035 (5)	0.0012 (4)
N1	0.0290 (7)	0.0187 (6)	0.0208 (7)	0.0034 (5)	0.0040 (5)	0.0011 (5)
N2	0.0263 (7)	0.0186 (6)	0.0202 (7)	0.0021 (5)	0.0023 (5)	0.0004 (5)
N3	0.0183 (6)	0.0140 (6)	0.0176 (6)	0.0004 (5)	0.0011 (5)	0.0001 (4)
N4	0.0173 (6)	0.0154 (6)	0.0172 (6)	−0.0005 (4)	−0.0001 (5)	0.0022 (4)
N5	0.0308 (7)	0.0171 (6)	0.0248 (7)	0.0010 (5)	0.0001 (6)	0.0014 (5)
C1	0.0158 (6)	0.0181 (7)	0.0194 (7)	−0.0026 (5)	0.0001 (6)	−0.0021 (5)
C2	0.0194 (7)	0.0192 (7)	0.0162 (7)	−0.0024 (5)	0.0011 (5)	−0.0001 (5)
C3	0.0167 (7)	0.0165 (7)	0.0218 (7)	−0.0020 (5)	−0.0009 (6)	−0.0013 (5)
C4	0.0161 (6)	0.0196 (7)	0.0202 (7)	−0.0029 (5)	0.0042 (6)	−0.0042 (6)
C5	0.0180 (7)	0.0230 (7)	0.0167 (7)	−0.0054 (6)	0.0021 (6)	−0.0008 (6)
C6	0.0176 (7)	0.0187 (7)	0.0206 (7)	−0.0019 (6)	−0.0003 (6)	−0.0001 (5)
C7	0.0331 (9)	0.0328 (9)	0.0195 (8)	0.0028 (7)	0.0024 (7)	0.0065 (7)
C8	0.0359 (9)	0.0312 (9)	0.0293 (9)	0.0037 (7)	−0.0016 (7)	−0.0147 (7)
C9	0.0274 (8)	0.0205 (7)	0.0240 (8)	0.0011 (6)	0.0009 (6)	0.0031 (6)
C10	0.0180 (7)	0.0184 (7)	0.0163 (7)	−0.0036 (5)	0.0005 (5)	0.0005 (5)
C11	0.0187 (7)	0.0148 (7)	0.0214 (7)	0.0004 (5)	0.0022 (6)	−0.0008 (5)
C12	0.0162 (6)	0.0135 (6)	0.0200 (7)	−0.0012 (5)	0.0007 (5)	−0.0013 (5)
C13	0.0150 (6)	0.0171 (7)	0.0192 (7)	−0.0009 (5)	0.0022 (5)	0.0003 (5)
C14	0.0238 (7)	0.0159 (7)	0.0225 (7)	−0.0009 (6)	0.0032 (6)	−0.0014 (5)
C15	0.0269 (8)	0.0243 (8)	0.0199 (7)	−0.0032 (6)	0.0035 (6)	−0.0020 (6)
C16	0.0249 (7)	0.0254 (8)	0.0200 (7)	−0.0010 (6)	0.0000 (6)	0.0048 (6)
C17	0.0247 (7)	0.0169 (7)	0.0205 (7)	0.0009 (6)	−0.0004 (6)	−0.0023 (5)

### Geometric parameters (Å, °)

**Table:**

S1—C11	1.7275 (15)	C3—C4	1.396 (2)
S1—C12	1.7606 (14)	C4—C5	1.396 (2)
O1—C5	1.3607 (17)	C5—C6	1.393 (2)
O1—C7	1.4242 (19)	C6—H6	0.9500
O2—C4	1.3739 (17)	C7—H7A	0.9800
O2—C8	1.416 (2)	C7—H7B	0.9800
O3—C3	1.3616 (17)	C7—H7C	0.9800
O3—C9	1.4269 (18)	C8—H8A	0.9800
N1—C11	1.3092 (19)	C8—H8B	0.9800
N1—N2	1.4007 (17)	C8—H8C	0.9800
N2—C10	1.3225 (19)	C9—H9A	0.9800
N3—C11	1.3645 (18)	C9—H9B	0.9800
N3—C10	1.3739 (18)	C9—H9C	0.9800
N3—N4	1.3767 (16)	C12—C13	1.4637 (19)
N4—C12	1.3016 (18)	C13—C14	1.388 (2)
N5—C17	1.331 (2)	C13—C17	1.394 (2)
N5—C16	1.343 (2)	C14—C15	1.381 (2)
C1—C2	1.395 (2)	C14—H14	0.9500
C1—C6	1.396 (2)	C15—C16	1.377 (2)
C1—C10	1.466 (2)	C15—H15	0.9500
C2—C3	1.392 (2)	C16—H16	0.9500
C2—H2	0.9500	C17—H17	0.9500
			
C11—S1—C12	87.47 (7)	O2—C8—H8B	109.5
C5—O1—C7	117.36 (12)	H8A—C8—H8B	109.5
C4—O2—C8	113.05 (11)	O2—C8—H8C	109.5
C3—O3—C9	116.95 (11)	H8A—C8—H8C	109.5
C11—N1—N2	105.24 (12)	H8B—C8—H8C	109.5
C10—N2—N1	109.42 (12)	O3—C9—H9A	109.5
C11—N3—C10	105.84 (12)	O3—C9—H9B	109.5
C11—N3—N4	118.28 (12)	H9A—C9—H9B	109.5
C10—N3—N4	135.86 (12)	O3—C9—H9C	109.5
C12—N4—N3	107.34 (11)	H9A—C9—H9C	109.5
C17—N5—C16	117.03 (13)	H9B—C9—H9C	109.5
C2—C1—C6	121.46 (13)	N2—C10—N3	107.93 (12)
C2—C1—C10	120.62 (13)	N2—C10—C1	125.37 (13)
C6—C1—C10	117.88 (13)	N3—C10—C1	126.53 (13)
C3—C2—C1	118.93 (13)	N1—C11—N3	111.57 (13)
C3—C2—H2	120.5	N1—C11—S1	138.90 (12)
C1—C2—H2	120.5	N3—C11—S1	109.52 (10)
O3—C3—C2	124.89 (13)	N4—C12—C13	122.53 (13)
O3—C3—C4	114.79 (13)	N4—C12—S1	117.39 (11)
C2—C3—C4	120.32 (13)	C13—C12—S1	120.07 (10)
O2—C4—C5	120.23 (13)	C14—C13—C17	118.08 (14)
O2—C4—C3	119.58 (13)	C14—C13—C12	121.19 (13)
C5—C4—C3	120.15 (13)	C17—C13—C12	120.72 (13)
O1—C5—C6	124.69 (14)	C15—C14—C13	118.68 (14)
O1—C5—C4	115.13 (12)	C15—C14—H14	120.7
C6—C5—C4	120.18 (13)	C13—C14—H14	120.7
C5—C6—C1	118.96 (14)	C16—C15—C14	119.00 (14)
C5—C6—H6	120.5	C16—C15—H15	120.5
C1—C6—H6	120.5	C14—C15—H15	120.5
O1—C7—H7A	109.5	N5—C16—C15	123.50 (14)
O1—C7—H7B	109.5	N5—C16—H16	118.3
H7A—C7—H7B	109.5	C15—C16—H16	118.3
O1—C7—H7C	109.5	N5—C17—C13	123.69 (14)
H7A—C7—H7C	109.5	N5—C17—H17	118.2
H7B—C7—H7C	109.5	C13—C17—H17	118.2
O2—C8—H8A	109.5		
			
C11—N1—N2—C10	0.12 (16)	N4—N3—C10—C1	3.5 (2)
C11—N3—N4—C12	0.04 (16)	C2—C1—C10—N2	−178.36 (14)
C10—N3—N4—C12	−178.10 (15)	C6—C1—C10—N2	−0.5 (2)
C6—C1—C2—C3	−0.4 (2)	C2—C1—C10—N3	−3.7 (2)
C10—C1—C2—C3	177.37 (13)	C6—C1—C10—N3	174.15 (13)
C9—O3—C3—C2	−5.2 (2)	N2—N1—C11—N3	0.27 (16)
C9—O3—C3—C4	175.44 (12)	N2—N1—C11—S1	−179.02 (14)
C1—C2—C3—O3	−178.33 (13)	C10—N3—C11—N1	−0.54 (16)
C1—C2—C3—C4	1.0 (2)	N4—N3—C11—N1	−179.19 (12)
C8—O2—C4—C5	90.63 (17)	C10—N3—C11—S1	178.96 (9)
C8—O2—C4—C3	−91.79 (17)	N4—N3—C11—S1	0.31 (15)
O3—C3—C4—O2	0.86 (19)	C12—S1—C11—N1	178.89 (18)
C2—C3—C4—O2	−178.51 (13)	C12—S1—C11—N3	−0.40 (10)
O3—C3—C4—C5	178.45 (13)	N3—N4—C12—C13	−179.41 (12)
C2—C3—C4—C5	−0.9 (2)	N3—N4—C12—S1	−0.38 (14)
C7—O1—C5—C6	−1.6 (2)	C11—S1—C12—N4	0.48 (11)
C7—O1—C5—C4	178.36 (13)	C11—S1—C12—C13	179.54 (12)
O2—C4—C5—O1	−2.16 (19)	N4—C12—C13—C14	−161.57 (13)
C3—C4—C5—O1	−179.73 (12)	S1—C12—C13—C14	19.42 (18)
O2—C4—C5—C6	177.85 (13)	N4—C12—C13—C17	18.6 (2)
C3—C4—C5—C6	0.3 (2)	S1—C12—C13—C17	−160.41 (11)
O1—C5—C6—C1	−179.70 (13)	C17—C13—C14—C15	−1.2 (2)
C4—C5—C6—C1	0.3 (2)	C12—C13—C14—C15	178.97 (13)
C2—C1—C6—C5	−0.2 (2)	C13—C14—C15—C16	0.3 (2)
C10—C1—C6—C5	−178.05 (13)	C17—N5—C16—C15	−0.9 (2)
N1—N2—C10—N3	−0.46 (16)	C14—C15—C16—N5	0.9 (2)
N1—N2—C10—C1	175.04 (13)	C16—N5—C17—C13	−0.1 (2)
C11—N3—C10—N2	0.60 (15)	C14—C13—C17—N5	1.1 (2)
N4—N3—C10—N2	178.89 (14)	C12—C13—C17—N5	−179.02 (13)
C11—N3—C10—C1	−174.83 (13)		

### Hydrogen-bond geometry (Å, °)

**Table:**

*D*—H···*A*	*D*—H	H···*A*	*D*···*A*	*D*—H···*A*
C2—H2···N4	0.95	2.40	3.0869 (19)	129
C9—H9A···N1^i^	0.98	2.60	3.576 (2)	171
C8—H8C···N5^ii^	0.98	2.63	3.573 (2)	161
C14—H14···N2^iii^	0.95	2.57	3.410 (2)	148

Symmetry codes: (i) −*x*+1, −*y*+1, −*z*+1; (ii) *x*, −*y*+3/2, *z*+1/2; (iii) *x*, −*y*+1/2, *z*−1/2.
